# Pushing the Limits of Transcatheter Edge-to-Edge Repair: How Small Can the Mitral Valve Area Go?

**DOI:** 10.1016/j.shj.2025.100782

**Published:** 2025-12-09

**Authors:** Gaspard Suc

**Affiliations:** Cardiology Bichat, AP-HP, UMRS1148, INSERM, Université Paris Cité, Paris, France

Mitral transcatheter edge-to-edge repair (m-TEER), initially reserved for patients contraindicated for surgery, has now been elevated to a Class IIa recommendation in the 2025 European Society of Cardiology Valvular Heart Disease Guidelines for high-surgical-risk patients with suitable anatomy.^1^ Procedural success, as defined by Mitral Valve Academic Research Consortium, requires achieving meaningful mitral regurgitation (MR) reduction—ideally to mild or less—while avoiding significant mitral stenosis, namely an effective orifice area ≥1.5 cm^2^ and a transmitral pressure gradient (TMPG) < 5 mmHg.^2^, ^3^, ^4^ Because the technique approximates the anterior and posterior leaflets, m-TEER inevitably reduces the mitral valve area (MVA), with prior studies showing an average ≈ 50% decrease in MVA immediately after clip implantation.^5^ Despite this predictable reduction, the safety and outcomes of m-TEER in patients who already have a small baseline MVA remain insufficiently characterized.

In this context, Chloé Khars et al. performed a retrospective single-center analysis of MitraClip procedures conducted between 2014 and 2022, focusing on patients with a baseline MVA <4.0 cm^2^.^6^ The study evaluated procedural success as well as short- and long-term clinical outcomes in this anatomically high-risk subgroup. Among 305 patients with available data, 22% had an MVA <4.0 cm^2^. Baseline TPMGs were similar, while moderate or severe mitral annular calcification (MAC) was more frequent in the small-MVA group. This group received fewer clips than the non-small-MVA group (MVA ≥ 4 cm^2^). Postprocedural TMPGs were modestly higher in patients with MVA <4.0 cm^2^, although the proportion with TMPG >5 mmHg did not differ significantly. Importantly, 2-year mortality, heart failure hospitalization, and composite outcomes were comparable across MVA categories, with neither MVA nor New York Heart Association class predicting events in multivariable analysis. Notably, indexed MVA demonstrated poor performance in predicting elevated TMPG (>5 mmHg) at discharge, with an area under curve of 0.41. The authors should be congratulated for exploring a clinically important and relatively understudied anatomical subset; nonetheless, several points deserve consideration.

First, defining a “small” MVA as <4.0 cm^2^ is open to debate. While Hausleiter et al.^7^ and the Endovascular Valve Edge-to-Edge Repair Study II framework proposed >4.0 cm^2^ as ideal for m-TEER, contemporary experience supports a more nuanced continuum, where 3.5 to 4.0 cm^2^ is complex but feasible, 3.0 to 3.5 cm^2^ very challenging, and <3.0 cm^2^ often unsuitable. A binary 4.0 cm^2^ threshold may therefore be conservative, and although the authors explored a 3.5 cm^2^ cutoff, that subgroup analysis was limited to patients already within the small-MVA cohort and was likely underpowered. While 3D transesophageal echocardiography planimetry provides the most anatomically accurate assessment of MVA, its acquisition remains technically demanding—particularly in the presence of heavy MAC or complex leaflet restriction—which likely explains why a substantial proportion of screened patients lacked a measurable MVA.^8^^,^^9^ In this setting, it is reasonable to consider whether baseline TPMG could serve as a more reproducible surrogate for the risk of postprocedural stenosis. The limitation, of course, is that TPMG is strongly flow- and MR-dependent: severe MR increases diastolic inflow, artificially elevating the gradient, which typically decreases once MR is reduced by TEER. However, an integrated Doppler approach combining TPMG, heart rate, and the slope of deceleration (pressure half-time) can provide a more physiologically grounded estimate of true stenotic risk, particularly in experienced hands.^10^ This multiparametric assessment is commonly used in clinical practice when planimetry is suboptimal or discordant and has been shown to correlate reasonably with anatomical MVA.

Another point of interest is the absence of outcome differences among patients with MVA <4.0 cm^2^ according to the presence of MAC. Given the well-established associations between MAC, adverse prognosis, chronic kidney disease, and cardiovascular risk clustering, these neutral findings probably reflect limited statistical power rather than a genuine lack of prognostic relevance.^11^^,^^12^ Furthermore, the analysis included only patients who ultimately underwent m-TEER, without considering patients deemed unsuitable; including screened-but-excluded patients would have provided a more complete view of anatomical boundaries and selection thresholds.

The observation that 68% of patients with MVA <4.0 cm^2^ received a single clip compared with 54% in the larger-MVA group also deserves attention. Although conservative clipping may reduce the risk of iatrogenic stenosis, it may predispose to late MR recurrence, a concern supported by previous studies demonstrating single-clip implantation as an independent predictor of recurrent MR, particularly in degenerative MR where progression of prolapse or suboptimal initial clip spacing often drives recurrence.^13^^,^^14^ Finally, all patients in this study were treated with MitraClip, without representation of the PASCAL device (Edwards Lifesciences, Irvine, California). PASCAL may theoretically offer advantages in anatomies at risk of elevated TPMG due to its broad leaflet-capture design and central spacer, although observational studies and meta-analyses have shown only modest differences in gradients, and the randomized Edwards PASCAL TrAnScatheter Valve RePair System Pivotal Clinical Trial did not demonstrate clinically meaningful differences in postprocedural stenosis between devices.^15^^,^^16^

In conclusion, this study provides reassuring data supporting the safety of m-TEER in patients with MVA ≤4.0 cm^2^ but also highlights the limitations of current anatomical thresholds. The most clinically challenging zone is not at 4.0 cm^2^ but rather <3.5 cm^2^—and especially <3.0 cm^2^—where the competing risks of residual MR and iatrogenic mitral stenosis converge. These patients, frequently burdened by significant MAC and complex leaflet restriction, represent the subgroup in whom TEER decision-making is most delicate yet least guided by high-quality evidence. As surgical options are often limited in severe calcified mitral valve disease,^17^ refining anatomical selection criteria, validating more reproducible markers of stenosis risk such as TPMG, and evaluating newer devices—including PASCAL and emerging transcatheter mitral valve replacement platforms should be prioritized.^18^ Ultimately, improving precision in patient selection will be essential to ensure the safe and appropriate expansion of TEER into this last frontier of complex mitral valve disease.

## Funding

The author has no funding to report.

## Disclosure Statement

G. Suc received a grant from the Federation Francaise de Cardiologie and Abbott.
